# Connecting authors with readers: what makes a good review for the *Korean Journal of Women Health Nursing*

**DOI:** 10.4069/kjwhn.2023.02.23

**Published:** 2023-03-31

**Authors:** Hyun Kyoung Kim

**Affiliations:** Department of Nursing, Kongju National University, Gongju, Korea

Thanks to its dedicated readers and reviewers, the *Korean Journal of Women Health Nursing* (KJWHN) has been indexed in Scopus, PubMed Central, and Emerging Sources Citation Index. After KJWHN was listed in major citation indices in a short time, the number of authors submitting their research to KJWHN has increased worldwide. The editors of KJWHN appreciate the authors who have submitted their manuscripts and the readers who have shown interest in KJWHN. The reviewers of KJWHN are striving for more transparent and professional reviews. From my perspective as an editor and a reviewer, this editorial presents opinions about good reviews that would facilitate the further development of KJWHN as a professional academic journal. What makes a good review? It may not be simply a matter of reading submitted manuscripts and providing opinions on them. Instead, a good review would help improve the quality of manuscripts by considering how readers will respond to them. The reviewer’s role involves reflecting the perspectives of both readers and authors on the thoughts and ideas in the manuscript and connecting both perspectives. This editorial aims to explain the role of reviewers, the characteristics of a good review, and what reviewers frequently miss or are likely to miss. This editorial will be helpful to authors who submit their manuscripts and reviewers who strive to review manuscripts to enhance the development of KJWHN into a top-tier academic journal.

## Role of reviewers

### Reviewing the scope of the topic

Since KJWHN covers research on women and their health, pre-screening may be restricted if the research topic is not about women. Likewise, manuscripts that do not deal with health, nursing, medical care, or well-being do not fit the journal’s scope. Although the scope of the journal may seem broad, many manuscripts that do not match its scope are being submitted. Therefore, authors must check the aims and scope of the journal in advance. Given the reality that interdisciplinary convergence studies are preferred, if a manuscript with a creative topic contains topics partially related to women or health, it can be reviewed. The ideal article would be a future-oriented manuscript with up-to-date trends and novelty that can contribute to women’s health, and a revolutionary article can be written on a traditional topic that has been researched repeatedly if it presents a new perspective [1]. If authors have any inquiries about the scope, please do not hesitate to contact the editorial office via email (kjwhn@kjwhn.org).

### Peer review

The core of a review is giving valuable comments to the authors. A review serves as a bridge connecting readers and authors. Reviewers are partially responsible for flawless publications. Furthermore, reviewing excellent manuscripts can help reviewers broaden their own horizons, gain ideas, and grow as scholars [2]. Therefore, reviewers should help authors improve manuscripts’ quality to make them robust, logical, and valuable articles that provide insights to readers. This is particularly important because KJWHN, as the official journal of the Korean Society of Women Health Nursing, fosters the development of studies, research, practice, and education in women’s health nursing in Korea and reflects the level of women’s health nursing. High-quality peer review is, therefore, key in order for KJWHN to share scientific and evidence-based knowledge with readers, as well as to develop as a world-class academic journal. In studies dealing with real-world aspects of women’s health, reviewers can comment on providing a theoretical framework or a conceptual model. In addition, reviewers should investigate global issues of women’s health and review manuscripts with the goal of enabling creative research to stimulate practice. Reviewers can request authors to cite international and recent studies as references.

### Decision-making

The reviewers of KJWHN make decisions by transparently choosing one of four options (reject, major revision, minor revision, and accept) in the electronic submission system. The decisions of KJWHN are made by the comprehensive judgment of two reviewers and one statistical reviewer. Each of the three reviewers conducts a blind review. If the decisions of reviewers are different, an additional reviewer can be selected to provide an opinion [3]. Since KJWHN recommends two weeks for review, it is important to review manuscripts in a timely manner. Although prompt decision-making is important for authors, reviewers experience a dilemma because an in-depth review is time-consuming. Editors support and help the review process, but do not intrude upon the inherent rights of reviewers. Editors also participate in the initial review and evaluation of manuscripts after review and present opinions to the authors. However, reviewers’ decisions are the primary consideration and are the key to publication. In KJWHN, two associate editors and one editor-in-chief participate in decision-making and the final review of manuscripts.

## Characteristics of a good review

### Politeness

Since peer reviewers are experts invited to review a manuscript, rather than critics, they must maintain an objective attitude toward the manuscript. There are occasional cases of severe criticism or assertive devaluations, but polite terms should be chosen even if a manuscript has substantial room for improvement. In order to write a reader-friendly review, one must change one’s viewpoint to evaluate whether the manuscript is easy to read from the reader’s perspective [1]. Since women’s health is always changing and developing, even top-tier experts should admit that there are areas they do not know and look at the manuscripts with an open attitude. A humble attitude comes from respecting authors and acknowledging the uniqueness of another scholar’s research. It is also important for authors to use thoughtful, constructive, scientific, academic, and clear language.

### Expanding expertise

Review is a communication process accompanying new learning and investigation. If a manuscript presents different opinions from those of the reviewers themselves, it is necessary to review the literature to see if there are new discoveries other than the existing knowledge [4]. Since analysis methods, new statistical techniques, and digital technologies have rapidly changed during the coronavirus disease 2019 pandemic, reviewers should be careful to avoid the extremes of excessive acceptance or rejection. Reviewing manuscripts provides a good opportunity for reviewers to expand their expertise and horizons. If a reviewer is requested to review a manuscript on a topic that they consider to be beyond their domain of expertise, it is possible to refuse the review request, and a second reviewer can be selected. Reviewers critically evaluate the structure and content of a manuscript, and, simultaneously, they can encourage authors to make better revisions. KJWHN conducts workshops to improve the competencies of reviewers and editors every year. The workshop videos for reviewers can be watched on the KJWHN homepage after logging into the e-submission system [5].

## What reviewers are likely to miss

### Structure of the review

Reviews should be written in detail, and it is helpful to plan the structure of a review in advance [3]. First, one should express appreciation to the authors for the submission and provide a complete sentence containing a general review of the manuscript. Then, very specific opinions should be presented and numbered to match the structure of the manuscript. Indicating page and line numbers will help authors clearly identify the issues pointed out in the review. Common grammatical mistakes include subject-verb disagreement, long sentences (more than three lines), single-sentence paragraphs, and misspellings. A reviewer who points out these mistakes should also write comments without grammatical errors to build trust in the reviewer. Therefore, it is recommended to double-check the comments after writing them; one can take some time and keep the review as a draft, rather than clicking “submit” immediately after writing the review.

### Reviewers’ investigations

Reviewers have an obligation to check manuscripts for ethical issues. The editorial office of KJWHN conducts a plagiarism check using the Copy Killer (MUHAYU Inc., Seoul, Korea) and/or iThenticate programs (Turnitin, LLC. Oakland, CA, USA). Since the plagiarism rate is provided at the word level, identical content is not allowed except for specific methodological descriptions. Reviewers can also request authors to check the sources of graphics, figures, and tables for copyright issues and to mention in the manuscript whether the use of assessment tools has been approved by both the original authors and authors of the translated version. In order to check the originality of the manuscript, the data collection period, details of consent, and respect for the study participants should be checked in the Methods section. When checking statistics, both simple errors and the validity of the analysis should be assessed. In this regard, reviewers serve as gatekeepers, since readers are generally receptive to the findings reported in articles that they consider having passed rigorous peer review [2]. One point that reviewers frequently miss is that many authors state that their studies are descriptive studies when they conduct a regression analysis, but a correlational design study would be appropriate. The articles published in KJWHN do not have a conclusion subheading so conclusions should be described in the last paragraph of the Discussion section. The Discussion section should be written in an order that starts with the most crucial research purpose in detail and then discusses the other aims of the study. Authors tend to write excessively short English abstracts, and reviewers should request authors to write the English abstract in about 240 to 250 words per the journal guidelines. It is helpful for authors if reviewers suggest specific items to be described in greater depth in the abstract, such as research tools or the data collection period. If authors write an ambiguous summary statement, the reviewer can request a detailed statement corresponding to the study results. The number of keywords is limited to five, and reviewers need to check whether they are MeSH (Medical Subject Headings) terms. The consistency of references with the main text should also be reviewed. Other common mistakes that KJWHN reviewers frequently miss are presented in Table 1.

### Review content

KJWHN only makes reviews available to authors and editors, and it does not disclose them to the public. Reviewers can be seen as playing the role of a hidden bridge. A responsible reviewer feels psychological responsibility for the results because the manuscript is often substantially changed through the review process. Since KJWHN has an open access policy and all manuscripts are open to all readers throughout the world permanently, reviewers are required to examine whether their influences are reasonable, helpful, and valid both in the present and in the future [1]. Reviews should not reveal the identities of reviewers, and information learned during the review process must be kept confidential. In addition, reviewers should confirm that the references are cited well and there are no missing parts in the description of research methods, as well as ensure that the manuscript is logical, flows well in terms of scientific context, and follows the EQUATOR guidelines with a comprehensive description [6].

Since reviewers are also potential authors of KJWHN, it is hoped that this editorial will also help reviewers to write high-quality manuscripts. We sincerely appreciate all our reviewers and hope that many reviewers will participate in the development of KJWHN in the future. Just as the quality of education cannot exceed the quality of teaching, the expertise of reviewers is crucial for improving the quality of manuscripts. We applaud our reviewers who are generous with their advice and time, and hope that together KJWHN can develop as a world-class academic journal.

## Figures and Tables

**Table 1.** Points to review in submissions to the *Korean Journal of Women Health Nursing* (KJWHN)

Number	Research part	Guidelines of KJWHN	Examples of frequent review errors in KJWHN
1	Aim of research	Clear description of the main purpose	Ambiguous description of the aims
2	Research design	Proper description of the research design	Descriptive or correlational study
3	Supporting statements	Support from sufficient evidence	Effect size evidence for calculating the sample size
4	Measurement	Present approval for the use of scales	Missing citations of translated measurement tools
5	Data collection	Describe mode of identifying, approaching, and recruiting participants	Missing description of the process for vulnerable subjects, such as students
6	Table	Avoid redundancy of tables and descriptions	Unnecessary table for some results
7	Discussion	Avoid repetitive descriptions	Redundancy between the Results and Discussion sections
		Discuss the theoretical implications	Missing implications about theoretical aspects
8	Study limitations	Specify the study limitations	Not reporting realistic limitations
